# Research on the relationship between college students' employability and IT skills training based on mixed research methods

**DOI:** 10.3389/fpsyg.2022.1054134

**Published:** 2022-12-06

**Authors:** Jiao Peng, Chao Deng

**Affiliations:** ^1^Data Science and Big Data Technology, Guangdong University of Science and Technology, Dongguan, China; ^2^Information Management and Information System, Guangdong University of Science and Technology, Dongguan, China

**Keywords:** IT skills training, employability, reliability analysis, factor analysis, *t*-test

## Abstract

In the Internet era, there is a mismatch between the skill demands of the IT industry in China and the employment prospects of computer science graduates. The COVID-19 pandemic has particularly highlighted co-existing challenges for industry recruitment and student employment. Many education institutions see IT skills training as a way to solve this conflict. The present paper employs a mixed methods approach to explore factors regarding computer science students' employability. The study used a questionnaire informed by an indepth literature review, full scale development theory and the theory of competency-based education. Reliability analysis and factor analysis methods were used to assess component reliability and structural validity. A total of 323 valid questionnaires were collected and subjected to mean and variance analyses to explore significant differences, including in terms of gender, in student employability. The results show that: (1) employability is divided into nine factors; (2) IT skills training can improve employability; (3) the employability level of computer science students who participate in IT skills training is high; (4) there are significant gender differences in professional ethics, scientific spirit and job-seeking skills, but no significant gender differences in humanistic qualities, computer cognition and operation skills, software design and development skills, system use and innovation skills, sustainable development capacity and teamwork skills. The identification of student employability factors can help education institutions to improve their training and can be used as a standard for students' self-evaluation and selfimprovement. The paper also provides suggestions for education institutions about how to set up IT skills training programmes to enhance students' future employment prospects in the IT industry.

## Introduction

Since the 18th National Congress of the Communist Party of China (CPC), the CPC Central Committee has attached great importance to employment and has always taken stabilizing and expanding employment as an important goal for the harmonious development of the economy and society. The popularization of higher education in the People's Republic of China has meant that the graduate employment market is increasingly competitive. According to the Ministry of Industry and Information Technology, in the next 5 years, the demand for IT-based jobs will result in 15 to 20 million vacancies. The most prominent talent gaps are in software development and network engineering. The demand for software development skills is increasing at a rate of 20% per year, with an annual increase of nearly one million vacancies. In 2017, there were 2.9 million vacancies in the country's IT industry, but only 1.05 million IT graduates. According to the Software and Information Technology Service Industry Development Plan 2016–2020, the economic value of China's domestic software and IT service industry will exceed eight trillion yuan in 2020, with nine million people needing to be employed in the industry. The Ministry of Education data shows that there were 8.74 million graduates with bachelor's degrees in 2020. There is therefore a big supply-and-demand gap in the IT industry, in which the demand far outweighs the supply.

IT education in the national higher education system currently cannot meet market demands for quality IT graduates. The COVID-19 pandemic has particularly highlighted the co-existence of recruitment challenges for industry and challenges for graduates in finding employment. There are two main reasons for this paradoxical scenario. First, the overall skill levels of graduates do not meet industry requirements. With the development of China's social economy, businesses have increasingly high requirements for the skill sets of workers. Students are required to possess not only professional knowledge and abilities but also high levels of non-professional skills such as communication, adaptability, professionalism, professional ethics, and ideological awareness (Feng, 2020). Second, students struggle in job interviews as they fail to understand interview questions and often have an inferiority complex. In the face of competition, they underestimate their abilities and lack the courage to actively compete for jobs (Zhao, 2015).

IT skills training programs often serve as an important bridge between education and industry. Through IT skills training, students' ability to meet employers' demands can be improved, their anxiety about employment can be reduced, their sense of self-efficacy can be enhanced, and the overall employment rate can be improved (Sun, 2018; Huang, 2019; Shi, 2020).

The present study addresses the following four research questions (RQs):

RQ1: What factors influence computer-related employment?
RQ2: Does IT skills training have a positive impact on employability?
RQ3: Are there significant differences in employability factors between particular groups of students?
RQ4: What suggestions can be made to develop IT skills training programs for higher education institutions to make them more suitable for computer-related work?

The study first analyses relevant literature before using a questionnaire to explore factors relating to IT students' employability. Analysis of the questionnaire data includes a focus on gender differences in competency factors. The study concludes by offering suggestions for the development of IT skills training programs.

## Literature review and hypotheses

Employability refers to the ability to obtain and maintain employment and to obtain new employment when necessary. Academic discussion of employability includes a focus on the ability of individuals to identify opportunities and obtain jobs and also focuses on individual knowledge, skills, and personal qualities (Dong, 2020). Rather than just involving a single ability, employability is thus the sum of many aspects including knowledge, technology, and outlook on life, values, and competitiveness. These factors directly determine the employment rate of students. Skills training helps strengthen students' awareness of employment, develops their professional and practical skills, and improves their overall attractiveness to employers (Zhou, 2018). Therefore, IT skills training is conducive to the development of both professional IT skills and a better understanding of the connection between skill sets and future job requirements (He, 2019).

### Employability

The concept of employability was first proposed in the 1950s. Chen (2019) believes that it refers to the ability of students to understand their ideal employment scenarios, meet their social needs, and realize their value in society through the learning of knowledge and skills. This has become the decisive factor in job-hunting success. The employability of students is a developmental concept characterized by dynamics and stages. Luo et al. (2017) believe that it refers to the skills needed to obtain employment and achieve employment goals.

### Factors of employability

There is no uniform specification of the factors of employability. The concept has gradually developed from an initial single dimension to two dimensions and then to three and four dimensions, and beyond. Wang (2014) stated that employability is a complex system that, in the main, involves 19 elements at three different levels: basic competence, job-seeking competence, and developmental competence, including skills before and after job-seeking. Mantz Yorke and Peter Knight developed the understanding, skills, efficacy beliefs, and metacognition (USEM) theory of employability. According to this theory, employability has four aspects: understanding of professional knowledge, having the professional and general skills required for work, possessing self-efficacy manifested as confidence, and metacognition ability, i.e., the ability to self-reflect on one's own learning and behavior. In China, researchers suggested that employability includes professional abilities, general skills, personal qualities, and career planning abilities (Huang et al., 2012, 2015, 2017, 2019). Shi (2012) believes that employability can be divided into five dimensions: job-seeking ability, professional ability, interpersonal ability, self-development ability, and emotion regulation ability. Dong (2017) believes that it can be divided into professional quality (overall level of professional skills), professional ability (having the most important core abilities), and general abilities (e.g., communication, learning, personal presentation, problem identification and solution, and personal management). These three areas are the most in demand by industry (Li, 2015). Wu (2019) believes that employability includes the basic ability (daily skills required for employment and career development), professional ability (knowledge, skills, and qualities for the professional field), personal characteristics (interpersonal skills, sense of responsibility, enterprising spirit, etc.), and social adaptability (career expectations, work experience, resilience). Wang et al. (2018) concluded through empirical research that the structure of college students' employability includes five dimensions: scientific and cultural quality, professional quality, social ability, physical and mental quality, and employability (Chen et al., 2020, 2021a,b, 2022). Based on this body of study, the present study proposes that the employability of computer science students may be affected by the following nine factors: professional ethics, scientific spirit, humanistic quality, computer cognition and operation ability, software design and development ability, system usage and innovation ability, sustainable development capacity, team capacity, and job application ability.

### IT skills training

IT vocational skills training can help students adapt to the needs of the economy and society, improve vocational skills, and better promote employment and industrial development (Pan, 2020). Driven by teaching reforms, to alleviate the employment challenges faced by students, educational institutions are gradually promoting IT skills training by connecting the professional knowledge mastered by students to corresponding employment opportunities. These institutions increasingly support students to develop their skills through vocational skills training in cooperation with industry (He, 2019). Shi (2014) proposed that vocational skills training has become a fundamental way to help graduates find jobs by improving their skills and employability. Therefore, IT skills training is an important way to develop skilled personnel. Students with both theoretical knowledge and practical skills are well-equipped to face challenges in a fiercely competitive job market and are able to find employment promptly (Ding et al., 2017).

### Professional ethics

Professional ethics refers to the ethical requirements and codes of conduct that people engaged in certain occupations should follow. It covers the relationships between practitioners and service recipients, between employers and employees, and between different occupations (Yin, 2019). In computer-related positions, professional ethics should be reflected in personal morality (e.g., showing dedication, honesty and trustworthiness, unity and cooperation, and innovation and learning) and in personal discipline (e.g., abiding by the law and by field-specific ethics and regulations, and upholding confidentiality) (Yang, 2018). During times of rapid IT development, computer security is very important. An influx of computer viruses and hackers has not only caused reputational and economic losses to individuals and organizations but has also threatened national economic and military security. Good professional ethics is not just encompassed by a code of conduct in professional activities but is also a prerequisite for good work (Huang, 2015).

The first hypothesis is given as follows:

H1: IT skills training has a positive impact on professional ethics.

### Scientific spirit

Scientific spirit refers to the combination of values, ways of thinking, and codes of conduct developed and accumulated by people engaged in long-term scientific practice. It is the core and soul of science, a unique spiritual temperament that captures the allure of science itself and is reflected in scientific activities. It is a subjective mental state that is gradually formed and constantly developed in the process of understanding the objective world (Zhou and Liao, 2012). The implication of the scientific spirit includes exploration, questioning, innovation, rationality, and empirical consciousness, which are necessary traits for innovation (Li et al., 2014).

The second hypothesis is given as follows:

H2: IT skills training has a positive impact on the scientific spirit.

### Humanistic quality

Humanistic qualities refer to the integration of various forms of education to guide students through their own practice *via* learning about cultural knowledge, knowledge of humanities, and social psychology. Han and Zhang (2015) believe that, by learning humanistic knowledge, students can expand their spiritual scope and ways of thinking to value life, attend to their physical and mental health, and treat people in society fairly. The development of humanistic qualities helps students acquire basic models of thinking, methods of observation, analysis, and problem-solving skills for education, employment, and life in general (Zong, 2016). With the iterative development of technology, humanistic training is needed for sustainable personal development. Therefore, most courses in IT skills training include courses to develop humanistic qualities (Xiao, 2020).

The third hypothesis is given as follows:

H3: IT skills training has a positive impact on humanistic qualities.

### Computer cognition and operation ability

As the computer is used in all walks of life, every student should be able to master comprehensive basic computer knowledge. Computer cognition and operation skills are the basic requirements for IT industry positions. Yang (2020) believes that these skills are mandatory requirements for contemporary society. The Office application is the most commonly used software, and the ability to operate this computer software is a necessary ability for work. Basic computer knowledge and the use of common software form the most basic components of computer skills training.

The fourth hypothesis is given as follows:

H4: IT skills training has a positive impact on computer cognition and operation ability.

### Software design and development ability

Software design and development occupies a core position in computing knowledge. It involves both the training of professional skills and creative thinking (Tian, 2018). At present, most students lack practical and project skills in software research and development. Therefore, Zheng (2018) believes that the development and structure of practice-based skills can be strengthened by establishing education-business cooperation, where the advantages of the industry can be fully utilized to nurture the skills required by society. Students may regularly visit businesses on field trips to combine their theoretical knowledge with practice to improve their design and planning skills.

The fifth hypothesis is given as follows:

H5: IT skills training has a positive impact on software design and development ability.

### System usage and innovation ability

Skills in system usage and innovation have become some of the important components of computer proficiency for today's digital economy. The demand for IT software skills is no longer limited to program design but increasingly focuses on innovation awareness and ability (Liu et al., 2021). Li et al. (2021) proposed to strengthen students' practice and encourage innovation through activities such as in-school practical training, business internships, discipline competition, innovative teamwork, and participation in scientific research projects. This helps to put innovation skills training into practice and to develop practical ability.

The sixth hypothesis is given as follows:

H6: IT skills training has a positive impact on system usage and innovation ability.

### Sustainable development capacity

The capacity for sustainable development is a comprehensive skill set that is essential for career development (Zhou, 2020). Educational institutions must be fully aware of the importance of sustainable development for students' vocational skills and should continuously develop their teaching, training models, and curricula so that students can develop a humble, enterprising attitude to work and a conscientious professional spirit. This is a crucial way in which students can cope with the pressure of a competitive employment environment (Huang, 2020).

The seventh hypothesis is given as follows:

H7: IT skills training has a positive impact on sustainable development capacity.

### Team capacity

In recent years, teamwork has become an important component of recruitment assessment and businesses reject applicants who cannot cooperate with and integrate into a new team. This means that today's graduates must not only possess professional skills but also have a keen sense of team spirit and teamwork (Wang et al., 2020). Software development projects in the IT industry are systematic, so it is necessary to build a team of an appropriate size, clarify the standards to be followed, continually optimize the development process through management, and improve the overall software development levels of the team. Attention should be paid to communication and collaboration among software developers (Huang, 2015). In the process of software development, teamwork will achieve better results (Wu, 2021). At present, most education institutions are updating their curricula to adopt methods such as group discussion and teamwork, which can greatly improve the teamwork skills of students (Liu and Liu, 2016).

The eighth hypothesis is given as follows:

H8: IT skills training has a positive impact on teamwork capacity.

### Job application ability

Job application skills are key to achieving successful employment. In IT skills training, lectures from industry celebrities and human resource experts' feedback can help students fully realize their professional attributes and values and support them to develop a strong work ethic and sense of social responsibility. To simulate recruitment scenarios, mock job fairs and interviews can be organized to improve students' ability to deal with these unfamiliar situations (Guo, 2015).

The ninth hypothesis is given as follows:

H9: IT skills training has a positive impact on job application ability.

### Control variables

Psychological and behavioral studies showed that female students have higher emotional intelligence (EQ) and language skills than male students, which makes them more skilled at interpersonal communication and helps promote feelings of trust, security, and respect (Zou et al., 2021). Zhang and Gao (2011) suggested that academic performance reflects the degree of professional knowledge and skills of students and is the key evaluative index. The academic performance of female computer science students is better than male students in both theory and practice; knowledge of professional theory is particularly advantageous compared to design and practice. Male students show slightly better performance in comprehensive software development courses. Peng et al. (2019) also showed similar findings, in which female students display solid professional theoretical knowledge and strong practical operation abilities, work harder, and perform better academically.

The tenth hypothesis is given as follows:

H10: There are significant gender differences in employability.

## Conceptual model

**Figure 1:**
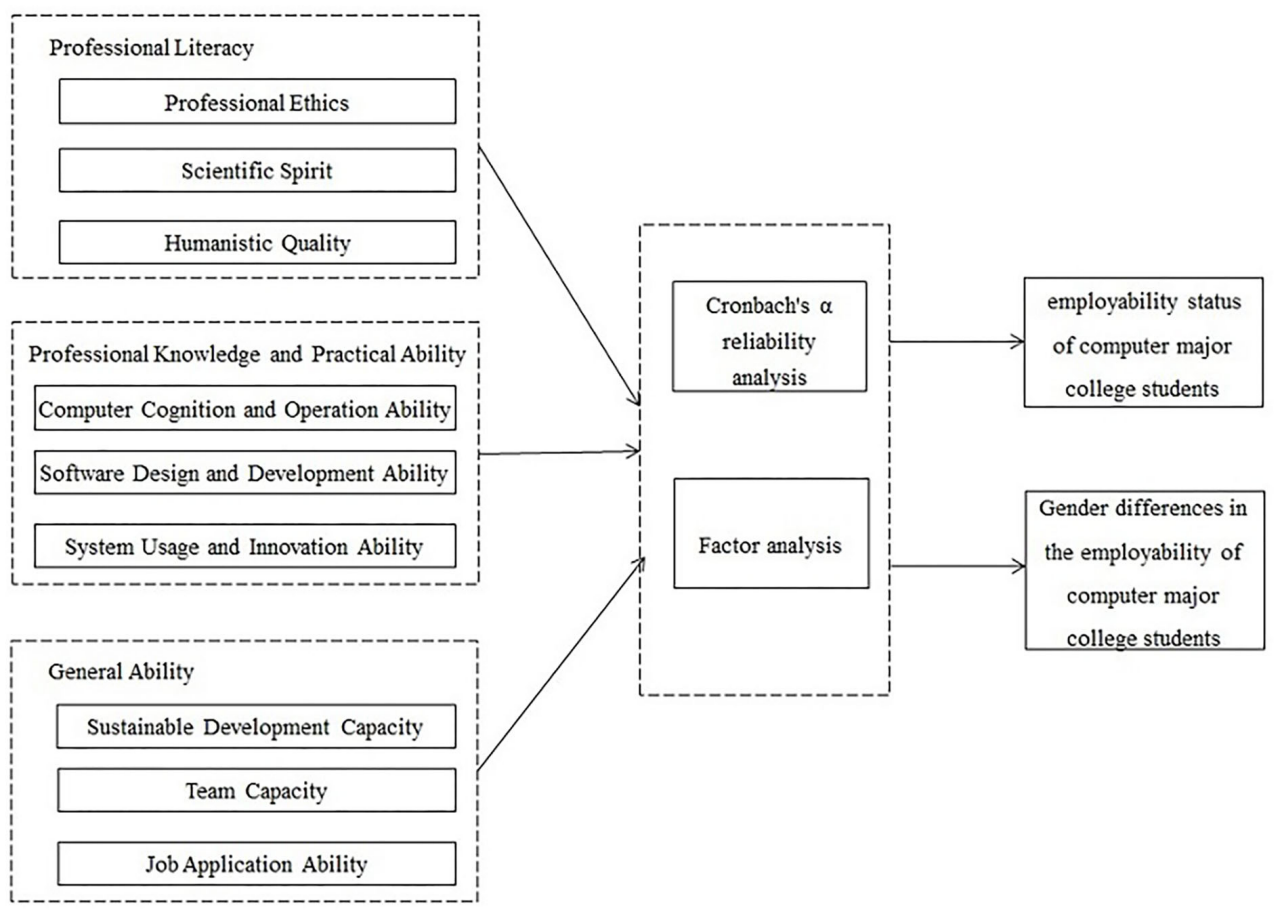
Conceptual model.

By drawing on development theory and quality-oriented education, the present study developed a questionnaire to explore the factors that constitute the employability of computer science students. Cronbach's α reliability analysis and factor analysis were used to measure the reliability and construct validity of questionnaire data and empirical tests. The means, variance analysis, and *t*-tests were used to analyze employability status and gender differences (see Figure 1). The results can help to clarify future training needs to improve employment outcomes.

## Research methods

Qualitative methods were used to review the literature regarding employability, IT skills training, and employability factors, and relevant theories. Quantitative methods were used to analyze the numerical questionnaire data *via* SPSS statistical software. Reliability analysis and hierarchical factor analysis were used to measure the reliability and construct validity of the components of employability.

The research took place in Dongguan and Guangzhou. Random sampling was used to select public undergraduate colleges, public junior colleges, private undergraduate colleges, and independent colleges. A sample size of 350 students was used, based on the Slovin formula, with each school's sample size allocated in proportion to the total number of students. Information on participants is provided in Appendix Table A.

Permission and assistance were sought from the relevant authorities to conduct the survey. The researchers designed and developed a questionnaire named “IT Skills Training Programme for Computer Related Employment”.

The researchers introduced the purpose of the study and the content of the questionnaire to school administrators by telephone or email and then requested permission to administer the questionnaire in the school. The questionnaire was distributed to computer science students through the Questionnaire Star online survey platform. The researchers also visited the schools to give out questionnaires to participants. The data analysis sought to clarify the factors of employability, employability status, and gender differences. The results can be used to inform future training and can also serve as a standard for students' self-evaluation and self-improvement, which has important practical significance for improving employment outcomes.

## Instruments

Following a literature review, the study involved a pilot questionnaire survey. The questionnaire was then modified and improved before formal data collection was carried out. Data were checked and inputted into SPSS software for analysis.

The questionnaire was designed according to the index of the employability structure of college students majoring in IT (2017) and the index of the employability and influencing factors of graduates majoring in finance and economics (2020), combined with employability factors of computer science students. A four-part questionnaire was used. The first part collected demographic data; the second, third, and fourth parts collected participants' self-rated scores on factors related to their employability, including professional literacy, professional knowledge, practical ability, and general skills. The latter three parts were measured by using a five-point Likert scale, where 1 = “Strongly Disagree” and 5 = “Strongly Agree”.

After the preliminary design of the questionnaire was completed, the questionnaire was released *via* Questionnaire Star. Thirty students participated in the study. SPSS26 was used to test the reliability of the sample data and Cronbach's α coefficient was used to measure the reliability of various factors of employability.

Table 1 shows that Cronbach's α coefficient of each dimension of employability is between 0.943 and 0.966, and all of them are greater than 0.9, indicating that the reliability of each indicator is very good.

**Table 1:** Analysis of reliability of the employability questionnaire.

**Indicators**	**Cronbach's α coefficient**	**Item number**
Professional ethics	0.960	5
Scientific spirit	0.963	5
Humanistic quality	0.958	5
Computer cognition and operation ability	0.945	5
Software design and development ability	0.943	5
System usage and innovation ability	0.958	5
Sustainable development capacity	0.964	5
Team capacity	0.966	5
Job application ability	0.955	5

Factor analysis was used to measure the construct validity of the questionnaire. Table 2 shows the analysis of professional ethics as an example.

**Table 2:** Factor analysis results for professional ethics.

**Indicator**	**Item**	**Component**	**Communalities**	**Kaiser–Meyer–Olkin (KMO)**	**Cumulative variance**
Professional ethics	1	0.929	0.863	0.907	86.240%
	2	0.938	0.879		
	3	0.934	0.872		
	4	0.912	0.831		
	5	0.931	0.867		

The value of KMO is 0.907, which is suitable for factor analysis. The commonalities of the five items are all >0.5, indicating that the variable is explained to a high degree by the factor. The component scores of the five items are all >0.9, higher than the minimum standard of 0.5, which indicates that each analysis item should belong to the corresponding factor and that the convergence validity of the index is high. If the cumulative variance is higher than 50%, the extracted factors are acceptable. Table 2 shows that the cumulative variance is 86.24%, indicating that the extracted factors are acceptable and that the construct validity is good. The results showed that the indicator of professional ethics can be extracted as a common factor, which remains named “professional ethics”.

Factor analysis of other indicators was conducted in the same way, and common factors were extracted from selected indicators to judge whether specific items of each dimension of employability meet the test standards of structural validity.

The original and revised copies of the questionnaire were submitted to the researcher's adviser Dr. Vivian B. Titular for her approval. Registered Psychometricians Kristine Joy Bautista, Dr. Auxilie Aurora D. Salvosa, and Dr. Queenie R. Demillo also validated the questionnaire and approved the items, especially the indicators of professionalism, as appropriate for formal research. This indicated that the validity of each dimension of self-evaluation is good.

## Participants

The survey was conducted using an online questionnaire among students from five universities in Guangdong, China.

The sample size, calculated by Slovin's formula, was 350. The sample size from each school was allocated according to the proportion of the total number of students majoring in computer science in the class of 2018. Information about the participants is shown in Appendix Tables A, B.

Students were informed of the purpose of the survey and could choose whether or not to participate. The questionnaire was launched and ran live for 1 month. After data cleaning, 323 valid questionnaires were obtained; 226 (70%) were from male students and 97 (30%) from female students.

## Results

Table 3 shows the means and standard deviations for perceived levels of employability of computer science students currently attending IT skills training. The data were gender disaggregated and a *t-*test was used to measure whether there was a significant difference (0.05 level) between male and female students (Table 4).

**Table 3:** Levels of perceived employability of computer science students.

**Indicators**	**Mean**	**Verbal interpretation**	**Std. deviation**
Professional ethics	3.72	H	1.2886
Scientific spirit	3.68	H	1.2316
Humanistic quality	3.55	H	1.1984
Computer cognition and operation ability	3.49	H	1.1766
Software design and development ability	3.498	H	1.1884
System usage and innovation ability	3.554	H	1.1824
Sustainable development capacity	3.63	H	1.2178
Team capacity	3.634	H	1.211
Job application ability	3.582	H	1.1569

Key: 1.00–1.80 = Very Low (VL); 1.81–2.60 = Low (L); 2.61–3.40 = Average (A); 3.41–4.20 = High (H); 4.21–5.00 = Very High (VH).

**Table 4:** Significant gender differences of computer science students in employability factors.

**Factors**	**Gender**	**Mean**	***t*-value**	**df**	***p*-value**	**Decision**	**Interpretation**
Professional ethics	Men	3.6372	−1.852	321	0.065	Accept H0	No Significant Difference
	Women	3.9052					
Scientific spirit	Men	3.5761	−2.362	321	0.019^*^	Reject H0	Significant Difference
	Women	3.9031					
Humanistic quality	Men	3.5053	−1.226	321	0.221	Accept H0	No Significant Difference
	Women	3.6701					
Computer cognition and operation ability	Men	3.4814	−0.199	321	0.842	Accept H0	No Significant Difference
	Women	3.5072					
Software design and development ability	Men	3.3982	−2.586	321	0.010^*^	Reject H0	Significant Difference
	Women	3.7320					
System usage and innovation ability	Men	3.5257	−0.730	321	0.466	Accept H0	No Significant Difference
	Women	3.6227					
Sustainable development capacity	Men	3.5575	−1.714	321	0.088	Accept H0	No Significant Difference
	Women	3.7938					
Team capacity	Men	3.5726	−1.503	321	0.134	Accept H0	No Significant Difference
	Women	3.7794					
Job application ability	Men	3.5027	−2.038	321	0.042^*^	Reject H0	Significant Difference
	Women	3.7649					

* The correlation is strongly significant at the 0.05 level (two-tailed).

As can be seen from Table 3, the mean values of all nine factors are high. This shows that the IT skills training program has a positive effect on all employability factors. Hypotheses H1, H2, H3, H4, H5, H6, H7, H8, and H9 are therefore valid.

Table 4 shows the *p-*values for scientific spirit, software design and development skills, and job application skills are all < 0.05, indicating that there are significant gender differences in these three factors. The *p*-values for professional ethics, humanistic qualities, computer cognition and operation skills, system usage and innovation skills, sustainable development capacity, and teamwork skills are all >0.05, indicating that there are no significant gender differences in these six factors.

## Discussion

### Composition of employability of computer science students

Researchers believe that employability is composed of professional ability, general skills, personal qualities, and career planning ability. Professional ability refers to the skills and knowledge acquired by students in the process of learning. General skills are those generic abilities most valued by the industry, including communication, learning, personal presentation, problem identification and solution, and personal management (Li, 2015). From the perspective of psychology, employability includes four basic dimensions, professional ability, employment personality, professional image, and personality compatibility (Gong and Cai, 2018). From the cognitive perspective, employability indicators include four dimensions of knowledge mastery, cognitive skills, organizational competence skills, and professional attitude (Song, 2017). In the present study, computer science students' employability were divided into professional ethics, scientific spirit, humanistic qualities, computer cognition and operation skills, software design and development skills, system usage and innovation skills, sustainable development capacity, teamwork skills, and job application skills. These nine first-level indicators were further subdivided into 45 secondary indicators.

### IT skills training can improve employability of computer science students

The IT industry is currently experiencing a period of rapid development. This requires a large number of employees with computer skills, but the skills of computer science graduates currently do not meet industry requirements. For example, practical operation ability is weak, professional quality is low, and understanding is poor. Education institutions are restructuring their curricula to promote vocational skills training, but at present, skills training remains relatively unpopular, the content is not driven by business requirements, and there is no targeted training and improvement. It is, therefore, necessary to fully appreciate the importance of vocational skill training courses and reform and improve training (Xia et al., 2019). Skills training helps strengthen students' awareness of employment, develops their professional and practical skills, and improves their overall attractiveness to employers (Zhou, 2018). Therefore, a new training model is proposed based on vocational ability, guided by employment requirements, and facilitated by education-industry cooperation. Students should pay attention to theory and practice, and engage with businesses so that they can apply their theoretical knowledge in practice and enhance their practical ability, teamwork skills, and maintain continuous professional development (Wang, 2014). IT skills training is thus helpful for nurturing professional IT skills and for highlighting the relationship between skills and job requirements (Xu and Liu, 2019).

### Levels of employability factors of computer science students gained in the IT skills training programs

According to the above analysis, the index score of computer science students' employability is between 3.38 and 3.81. Composite mean values are all between 3.49 and 3.72, reflecting that employability is at a high level. The best performance is seen in professional ethics, with a value of 3.72, and the score for computer cognition and operation skills is relatively low, with a value of 3.49.

Good professional ethics are essential, but the current education system focuses more on professional knowledge and practical skills, and many students' moral concepts are not consistent with their professional abilities. Therefore, within IT skills training, the “Laws and Ethics of the IT Industry” course has been established to strengthen the development of professional ethics. There is also a joint partnership with the industry to provide internship opportunities for students to develop their professional ethics (Jia, 2021). Previous studies are consistent with the results of the present study and hypothesis H1 is valid.

Currently, college students are used to passively receiving knowledge and rarely show the ability to think independently or the courage to question (Meng, 2019). Zhao (2010) suggested that the development of students' scientific spirit should be included in any training plan so as to encourage truth-seeking, an innovative and critical spirit, and encourage students to devote themselves to scientific practice. Therefore, courses relevant to the development of scientific spirit are included in IT skills training, and students are actively encouraged to participate in scientific practice by taking alternative classes (Wang et al., 2012). Previous studies are consistent with the results of the present study and hypothesis H2 is valid.

The development of humanistic qualities helps students acquire basic models and methods for thinking, observation, analysis, and problem-solving (Zong, 2016). Zhou and Zheng (2021) found that computer science students had a narrow range of knowledge and lacked awareness of literature, art, and aesthetics. To nurture humanistic qualities, it is necessary to establish specific courses and integrate the development of artistic aesthetics into professional courses. Previous studies are consistent with the results of the present study and hypothesis H3 is valid.

The popularization of computers requires that every student should master basic computer knowledge. However, students' computer operation and application skills are limited and uneven (Yin, 2018). The main reason for this is that the theoretical courses in computer science are perceived as dull, meaning that students only develop a superficial understanding of aspects such as the history of the computer, system structure, and hardware and software composition (Du, 2020). Courses such as the application of Office software, the composition of a computer system, and computer assembly have been added to further enhance operation and application skills. Previous studies are consistent with the results of the present study and hypothesis H4 is valid.

Software design and development occupies a core position in the disciplinary knowledge of computer science. It not only includes the development of vocational skills but also requires creative thinking. Liu (2016) highlighted that a lack of relevant experience and skills among computer science students means that their attempts to develop software often result in bugs, which affect the quality of software development. Therefore, it is necessary to revise software design training to include three levels of content: basic level, comprehensive level, and practical application level (Guo et al., 2019). IT skills training programs containing skills training and integrated practice can enhance students' ability to design and develop software. Previous studies are consistent with the results of the present study and hypothesis H5 is valid.

Software design and innovation skills have become particularly important in the testing of comprehensive computer skills. The demand for IT software skills goes beyond program design ability to include attention to innovation awareness and skills (Liu et al., 2021). Computer science graduates currently lack professional and practical skills, the ability to analyze and solve problems and the ability to innovate and create, among others (Yu and Zhu, 2021). By improving practical teaching, curriculum design, small-scale software development, education-industry cooperation, and on-the-job learning, students' developmental and innovation skills can be gradually nurtured (Song, 2022). Relevant courses are included in the IT skills training program and engineers from business are employed as instructors. Previous studies are consistent with the results of the present study and hypothesis H6 is valid.

Sustainable development refers to the collection of knowledge, skills, attitudes, and values needed to successfully complete tasks and solve problems in the context of sustainable development challenges and opportunities in the real world (Gu, 2022). IT skills training is moving toward adopting a long-term development vision to determine vocational education training objectives that enable students to be able to continuously adapt to a changing world post-graduation (Guan and Li, 2022). Therefore, previous studies are consistent with the results of the present study, and hypothesis H7 is valid.

Today's graduates should not only possess professional skills but also have a strong sense of teamwork (Wang et al., 2020). At present, computer science degrees tend to focus on skills such as software development and overlook IT industry requirements for skills such as teamwork. This means that it is difficult for computer science students to find employment (OuYang et al., 2017). Software development projects in the IT industry are systematic and particular attention should be paid to communication and cooperation among software developers (Huang, 2015). The comprehensive project practice courses within IT skills training can greatly improve students' teamwork skills. Previous studies are consistent with the results of the present study and hypothesis H8 is valid.

Many computer science students apply for unsuitable positions and struggle during job interviews to show the full extent of their abilities. This is a waste of time and is also detrimental to their future employment and development (Ren, 2021). IT skills training can enhance students' job application skills by simulating real-world recruitment scenarios *via* mock job fairs and mock interviews (Guo, 2015). Therefore, previous studies are consistent with the results of the present study, and hypothesis H9 is valid.

In summary, the basic courses, specific skills training courses, and real-world projects offered in IT skills training have a positive impact on the employability of computer science students.

### Significant differences in employability among computer science students

The educational and teaching practice of psychology and sociology shows that there are significant gender differences in cultivating good behavior habits (behavior cultivation), with male students traditionally performing better than female students (Shi, 2015). However, the fairer distribution of today's modern education resources means that girls and boys now have the same learning opportunities. In addition, girls have a strong sense of independence and mature earlier than boys (Lin, 2013). The present study found no significant gender difference in work ethic, which is consistent with previous research.

Luo (2019) proposed that female students have a lower sense of innovation and entrepreneurship, a lack of subjective consciousness, and a lower ability for psychological control. Sun (2022) also proposed a lack of awareness of innovation and entrepreneurship. The present study reinforces these proposals as there were significant differences in scientific spirit between male and female students.

Cao (2020) highlighted that female students score higher than male students in terms of humanistic qualities, which may be related to gender differences in personality and preferred hobbies, as girls focus more on literature and art while boys are more attuned to current events. Xu (2021) also proposed that there is no significant gender difference in terms of humanistic qualities. The results of the present study are consistent with this prior research.

Zhang and Gao (2011) posited that the academic performance of female computer science students is better than male students in both theory and practice; understanding professional theory conveys a particular advantage, with design and practice conferring a lesser advantage. Male students perform slightly better in comprehensive software development. Peng (2014) also made similar claims and proposed that female students work harder and achieve greater academic success. Female students have stronger motivation and a sense of pride and show greater recognition of the importance of specialized courses and different learning goals with the ability to adjust their approach accordingly. Computer science involves a combination of theory and practice, focusing on software development and technology application. There is no significant gender difference in computer cognition and system use, but there is a significant difference in terms of software design and development skills (Dai and Hu, 2018). The results of the present study are consistent with previous literature.

The sustainable development capacity of male students is stronger than that of female students, but there is a high level of individual difference among male students, meaning that the group score is less stable than that of female students. There is no significant gender difference in sustainable development capacity (Cai, 2018). The results of the present study are consistent with previous literature.

Survey data analyzed by Sun et al. (2015) showed strong levels of independence in male students, whereas female students place more importance on teamwork. The teamwork score of female students is slightly higher than that of male students, but male students are more capable than female students in dealing with conflict. In general, there is no significant gender difference in teamwork skills, which is consistent with previous research.

There are many factors affecting students' job application skills, including communication skills (both linguistic and social) and personal presentation. There is no significant gender difference in the various dimensions of job application skills. Although female graduates' presentation skills are significantly superior to those of male graduates, this does not correspond to better job application outcomes. This may be related to gender discrimination in the job market (Wei et al., 2020). The results of the present study are consistent with previous research which highlights gender discrimination in the employment process.

## Conclusions

This study has used a literature review and a survey to explore the relationship between IT skills training and factors of employability of computer science students. Statistical analysis was conducted to assess significant gender differences. The results can help educational institutions to improve employability through training and can serve as a standard for students' self-evaluation and self-improvement. The study also provides suggestions for educational institutions to set up IT skills training programs.

The study offers the following conclusions:

(1) Employability of computer science students is determined by nine factors: professional ethics, scientific spirit, humanistic quality, computer cognition and operation ability, software design and development ability, system usage and innovation ability, sustainable development capacity, team capacity, and job application ability.
(2) The employability level of computer science students is relatively high. Performance in professional literacy and general abilities is relatively good, but performance in professional knowledge and practical abilities is poor.
(3) There are significant gender differences in professional ethics, scientific spirit, and job application ability, but there are no significant differences in humanistic quality, computer cognition and operation ability, software design and development ability, system use and innovation ability, sustainable development capacity, and team capacity.

### Limitations

Employability is divided into broad employability and core employability. This study has only considered core employability, which is related to the individual qualities of students themselves, including the ability to find a job, to complete the job, and to achieve good career development. The study has not considered broader external environmental factors.

The study used a quantitative approach, but due to the impact of the COVID-19 pandemic, travel was constrained in China and therefore other methodological approaches that may shed light on participants' psychological dynamics were not possible.

The study is limited to the academic year 2020–2021. The participants were all junior computer science students who had participated in the IT skills training program organized by their educational institutions and had a basic knowledge of Java language and website development.

The training content of the program mainly focused on the development of professional knowledge and practical abilities required by the IT industry. There is less focus on more general skills because educational institutions may be legally required to offer specific courses related to general employment guidance.

### Suggestions for improvement

The following suggestions are proposed to improve the employability of computer science students:

(1) Computer science students could actively participate in various practical activities organized by their institution, such as innovation and entrepreneurship competitions, social practice activities, and academic forums. This will allow them to obtain relevant national vocational qualification certificates and improve their practical skills.
(2) Teachers and trainers could strengthen their teaching skills, actively participate in basic teaching skills competitions, and continue to learn, improve, and innovate in their practice. This may be achieved by visiting scholarships to broaden their vision and increase their knowledge or by participating in short- or long-term practice in the industry to understand the needs of businesses and to strengthen the relationship between industry and education.
(3) Educational institutions could offer different IT skills training programs based on gender, particularly in terms of focus on scientific spirit, software design and development ability, and job application ability.
(4) Training organizers could plan their curricula effectively so that the content is aligned with market skill-set demands. Practical teaching should be geared toward the needs of employers so that there is a close connection between knowledge and practice. The practical skills of students should be strengthened, and the approach of “teaching, learning, doing, examination and evaluation” should be implemented.
(5) Businesses could provide more internship opportunities for students and make better connections between students on placement and postgraduate employment.
(6) This study has analyzed the composition of computer science students' employability, current employability status, and gender differences in employability. The training content focuses on software design and development. Future research should include an analysis of the employability of students studying different courses.

## Data availability statement

The original contributions presented in the study are included in the article/Supplementary material, further inquiries can be directed to the corresponding authors.

## Author contributions

JP and CD: conceptualization, funding acquisition, and supervision. JP: study design, conducting study, analysis, interpretation, writing—original draft preparation, review, and editing. All authors have read and agreed to the published version of the manuscript.

## Funding

This work was supported by Guangdong University of Science and Technology, Intelligent Computing Innovation and Application Research Platform, GKY-2020CQPT-2 and Research Project of Guangdong University of Science and Technology: Analysis and Research of College Students' Online Learning Behavior in the Environment of Big Data, GKY-2020KYZDK-11 and Product Recommendation Algorithm Optimization based on Spark Technology, GKY-2019KYYB-33.

## Conflict of interest

The authors declare that the research was conducted in the absence of any commercial or financial relationships that could be construed as a potential conflict of interest.

## Publisher's note

All claims expressed in this article are solely those of the authors and do not necessarily represent those of their affiliated organizations, or those of the publisher, the editors and the reviewers. Any product that may be evaluated in this article, or claim that may be made by its manufacturer, is not guaranteed or endorsed by the publisher.
