# Supplementary material for: Research on the relationship between college students' employability and IT skills training based on mixed research methods
Source: Front Psychol. 2022 Dec 6;13:1054134. doi: 10.3389/fpsyg.2022.1054134 (PMC9763573; doi:10.3389/fpsyg.2022.1054134)
Supplement: Supplementary file 2 [file Data_Sheet_1.pdf]

Appendix

Appendix A

Table A. Distribution of Participants

University	Total Number	Sample
**Public**		
Guangdong University of Technology	800	100
Dongguan Polytechnic	320	43
**Private**		
Guangdong University of Science and Technology	1200	150
City College of Dongguan University of Technology	460	57
**Total**	**2780**	**350**

Table B. Profile of Participants

University	Gender	Frequency	Percentage
Guangdong University of Technology	Male	70	70%
	Female	30	30%
Dongguan Polytechnic	Male	30	69%
	Female	13	31%
Guangdong University of Science and Technology	Male	105	70%
	Female	45	30%
City College of Dongguan University of Technology	Male	40	70%
	Female	17	30%

Appendix B

Table A Questionnaire: IT Skills Training Programme for Computer Related Employment

Part I. Profile of Participants

A. Gender

___ Male

___ Female

B. Major: _______________

Part II. Self-evaluation of Professional Literacy

Using the scale below, place a check (√) in the appropriate column to indicate your response.

Legend:

5 - Strongly Agree

4 - Agree

3 – Neither Agree nor Disagree

2 – Disagree

1 - Strongly Disagree

**Professional Ethics**	**5**	**4**	**3**	**2**	**1**
1. I give importance to the positions in IT industry very much.					
2. I can be very responsible to complete the IT work.					
3. I have the necessary values like honesty required in the IT industry.					
4. I can handle IT work in a professional manner.					
5. I abide by the laws and disciplines of the IT industry.					
**Scientific Spirit**	**5**	**4**	**3**	**2**	**1**
1. In the IT industry, I always make sure to undergo training to update my skills.					
2. In the work of computer related positions, it is important to develop my critical thinking skills.					
3. In the work of computer related position, I always hone my creativity.					
4. I always practice my problem-solving skills as it is essential in working in IT Industry					
5. I have an innate sense of curiosity about how things work which I find helpful in working in an IT Industry.					
**Humanistic Quality**	**5**	**4**	**3**	**2**	**1**
1. I have adequate knowledge about the historical components required by the IT industry.					
2. I have the literary knowledge required					

by the IT industry.					
3. I possess political qualities needed in the IT industry.					
4. I have knowledge about the legal aspects in the IT industry.					
5. I have the artistic knowledge required by the IT industry.					

Part III. Self-evaluation of Professional Knowledge and Practical Abilities

Using the scale below, place a check (√) in the appropriate column for your response.

Legend:

5 - Strongly Agree

4 - Agree

3 – Neither Agree Nor Disagree

2 – Disagree

1 - Strongly Disagree

**Computer Cognition and Operation Ability**	**5**	**4**	**3**	**2**	**1**
1. I have a background about the history of computers.					
2. I understand the system structure of a computer.					
3. I am knowledgeable about the parts of the hardware of a computer.					
4. I understand the software structure of a computer.					
5. I can assemble a computer unit properly.					
**Software Design and Development Ability**	**5**	**4**	**3**	**2**	**1**
1. I have knowledge about operating system.					
2. I understand the framework and techniques used in Web development.					
3. I am skilled in using SSM framework for software development.					
4. I can use Python and Java well.					
5. I can design web pages.					
**System Usage and Innovation Ability**	**5**	**4**	**3**	**2**	**1**

1. I can read and understand software instructions.					
2. I understand the constraints and interfaces used by the software.					
3. I can complete software installation.					
4. I can use different types of software skillfully.					
5. I can do routine maintenance on the software.					

Part IV. Self-evaluation of General Abilities

Using the scale below, place a check (√) in the appropriate column for your response.

Legend:

5 - Strongly Agree

4 - Agree

3 – Neither Agree Nor Disagree

2 – Disagree

1 - Strongly Disagree

**Sustainable Development Capacity**	**5**	**4**	**3**	**2**	**1**
1. I apply work ethics of IT industry.					
2. I have the ability to learn new knowledge about IT industry.					
3. I have the ability to apply specialized knowledge of computer.					
4. I have good interpersonal skills which can be used in this industry.					
5. I have the ability to do computer related work.					
**Team Capacity**	**5**	**4**	**3**	**2**	**1**
1. I have good interpersonal skills					
2. I can be involved in completing the business of the IT technical team					
3. In IT related tasks, I have the ability to plan and set the necessary steps for my team					
4. I have strong communication skills that I can use in communicating with my technical team					

5. I can work with the team to identify IT questions					
**Job Application Ability**	**5**	**4**	**3**	**2**	**1**
1. I am confident that I have the abilities needed in the IT Industry.					
2. I know where to search for job opportunities in the IT Industry.					
3. I have the ability to write resumes for IT industry positions.					
4. I have a certain ability to express myself that is essential in job application.					
5. I have a certain ability of self-promotion.					
